# Doing Things Differently: What It Would Take to Ensure Continued Access to Contraception During COVID-19

**DOI:** 10.9745/GHSP-D-20-00171

**Published:** 2020-06-30

**Authors:** Michelle Weinberger, Brendan Hayes, Julia White, John Skibiak

**Affiliations:** a Avenir Health, Washington, DC, USA.; b Global Financing Facility, Washington, DC, USA.; c Reproductive Health Supplies Coalition, Brussels, Belgium.

## Abstract

COVID-19 may fundamentally change women’s contraceptive use, meaning that the future we have been planning and procuring for, may not match these changes. In these unprecedented times, we must rethink how we link product and program in the short-term to ensure women’s changing needs are met.

## Key Messages

- COVID-19 may fundamentally change women’s contraceptive use, thus altering the range of supplies that will be required in the near term. Policy makers will need to consider country realities and explore service delivery adaptations to meet these changing needs. Existing data can quantify potential shift in contraceptive use to help inform decisions.
- Donors, policy makers, and program planners may need to revisit supply plans and the use of financing to ensure that contraception is effectively sustained.

Since the start of the coronavirus disease (COVID-19) pandemic, the family planning community has focused their attention on mitigating the devastating consequences of failing to meet women’s needs for contraception. Recent estimates by the Guttmacher Institute suggest that with even just a 10% decline in use of short-term and long-acting reversible contraceptives (LARC) across 132 low- and middle-income countries, unmet need for contraception would increase by 48.6 million women and lead to 15 million additional unintended pregnancies.^1^ That risk grows each day as reports come to light of clinic closures, the reduced mobile outreach services^2^^,^^3^ and declines in the number of clients attending even open clinics.^4^

To ensure women’s access to a full range of methods as well as removal services, we have seen calls from across the RH community to safeguard the integrity of existing service delivery systems and the supply chains that support them.^5^^,^^6^ These calls are critical and they are welcome. But in environments where these systems face pressure or cease to function altogether, different solutions are being proposed, such as those made by Nanda et al. in this issue of GHSP.^7^ They outline approaches such as minimizing family planning client-provider contact through use of telehealth and integration into other essential services (same-day postpartum family planning). They also consider extended use of LARCs, options for method switching, and changing dispensing guidelines in the event disruptions are encountered. Many of the suggestions in their piece have also been echoed elsewhere.^8^^–^^12^

COVID-19 is fundamentally changing the contraceptive landscape, and by extension, the ability of national programs to meet women’s immediate needs for contraception. The future for which we have been planning and procuring will not be, in all likelihood, the reality we see before us in the coming 12–18 months. It is a mistake, therefore, to call for supply chains to continue feeding programs with a mix of supplies that they may no longer be capable of delivering. COVID-19 is raising a host of important questions about the relationship between product and program. In these unprecedented times, we must rethink the ways we link products and programs to ensure continuity in women’s access to contraception.

To add context to these policy discussions, we have attempted to quantify potential shifts in contraceptive use that could result from some of the mitigation strategies we have outlined. We focus on the mitigation strategies most likely to reflect the availability of contraceptive options under COVID-19 to highlight the implications of such changes.

## WHAT MIGHT BE IN STORE FOR CONTRACEPTIVE USERS?

The combination of stay-at-home orders, overwhelmed health care systems, and simply fear of contagion will likely increase the demand for self-care, defined by the World Health Organization as^13^:

*The ability of individuals, families and communities to promote health, prevent disease, maintain health, and cope with illness and disability with or without the support of a health care provider*.

For contraception, self-care methods include contraceptive pills, condoms, patches, rings, emergency contraception, Standard Days Method, and potentially self-injection of subcutaneous depot medroxyprogesterone acetate (DMPA-SC). At the same time, there may be decreased demand for products that require face-to-face contact with a health care provider or may be more difficult to obtain, including intrauterine devices, implants, and provider-administered injections. These changes would run counter to recent trends in contraceptive use and public sector procurement. For example, in 2018, two-thirds of spending in the public sector family planning market was on implants (US$88 million) and injectables (US$65 million).^14^

Each country is facing COVID-19 from a different starting place: different method mixes among its current users, differing roles of the public and private sectors, different levels of stock on hand, and different supply chain barriers. And each country’s response will vary depending on how the pandemic unfolds and what choices women make about their continued contraceptive use.

Each country’s response will vary depending on how the COVID-19 pandemic unfolds and what choices women make about their continued contraceptive use.

We used data from the 2019 Commodity Gap Analysis^15^ to simulate how different strategies for mitigating the disruption of services caused by COVID-19 might play out in different countries. To do this, we created 2 scenarios (Table), each of which estimates the potential for method switching under different levels of COVID-19 related service disruption (Supplement includes detailed assumptions). These scenarios assume that all current users of a modern family planning method would continue to contracept with some degree of method switching. Some women may instead discontinue use in the short run. These scenarios are not meant to suggest what we think will or should happen but rather to provide a starting point for discussions by quantifying the programmatic implications of these policy changes and of the choices women may make about what methods to use.

**TABLE.** Summary of Assumptions Used for COVID-19 Disruption Scenarios^a^

**Current Contraceptive Method Used**	**What We Assumed Would Happen With Minimal Disruption to Services**	**What We Assumed Would Happen With High Disruption to Services**
LARC	Half of women due for removal would continue to use their method beyond the labeled duration. For the remaining, half of replacement use would still be LARC, with the rest distributed across injectables and other self-care methods.	Nearly all women due for removal would continue to use their method beyond the labeled duration. For the rest, replacement use would mostly consist of self-care methods.
Injectable (not self-administered)	Half of women would continue to access their reinjections, with some women shifting to self-injection. Remaining users would switch to other self-care methods.	Only a small share of women would continue to access reinjections, with some women switching to self-injection. Remaining users would switch to other self-care methods.
Pill	The vast majority of pill users would continue to use pills. Users may be given advanced provision of 6 or 12 cycles to limit their need to return.	The vast majority of pill users would continue to use pills. Users may be given advanced provision of 6 or 12 cycles to limit their need to return.
Condom	The vast majority of condom users would continue to use condoms. A small share would switch to other self-care methods.	The vast majority of condom users would continue to use condoms. A small share would switch to other self-care methods.

Abbreviations: LARC, long-acting reversible contraception.

a See Supplement for detailed assumptions.

**FIGURE 1.**
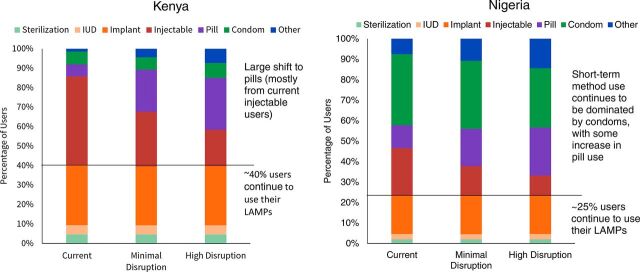
Potential Short-Term Changes in Contraceptive Method Mix During COVID-19 Disruptions in Kenya and Nigeria Abbreviations: IUD, intrauterine device; LAPM, long-acting and permanent method.

Although individual women may make similar choices with respect to self-care methods, the aggregate changes in method use, procurement needs, and cost would differ greatly among countries. Figure 1 compares changes in method mix in Kenya and Nigeria under the 2 scenarios.

In Kenya, around 40% of women are already using a long-acting or permanent method, with most using implants. Because implant use in Kenya has increased in recent years, only a small share of users would be due for implant removal/replacement in the coming months. Furthermore, based on evidence that many LARC methods can safely be used beyond their labeled duration,^16^^,^^17^ it is reasonable to assume that many users with a scheduled method replacement this year could remain protected from unintended pregnancy without an additional service during COVID-19 disruptions should they desire to continue using their method. Of course, when women do require a removal, efforts should be made to ensure safe access to services.

The largest segment of users in Kenya are injectable users—making up more than 45% of all modern contraceptive users. These women are likely to find themselves at greatest risk since continued use requires regular interaction with a health care provider. As disruptions increase, we assume that more of these women will switch to self-care methods, likely contraceptive pills, which offer similar protection to their current method of choice.

We can contrast this picture with Nigeria, where use of implants is much lower and use of condoms is higher. Although we also project increases in pill use in Nigeria, the magnitude of that increase is smaller. The overall high reliance on short-term methods in Nigeria means that more women are potentially susceptible to COVID-19 disruptions of those methods. For example, disruptions in condom supplies or price increases could have a large impact on contraceptive use in the country.

## WHAT COULD THESE CHANGES MEAN FOR SUPPLY NEEDS?

**FIGURE 2.**
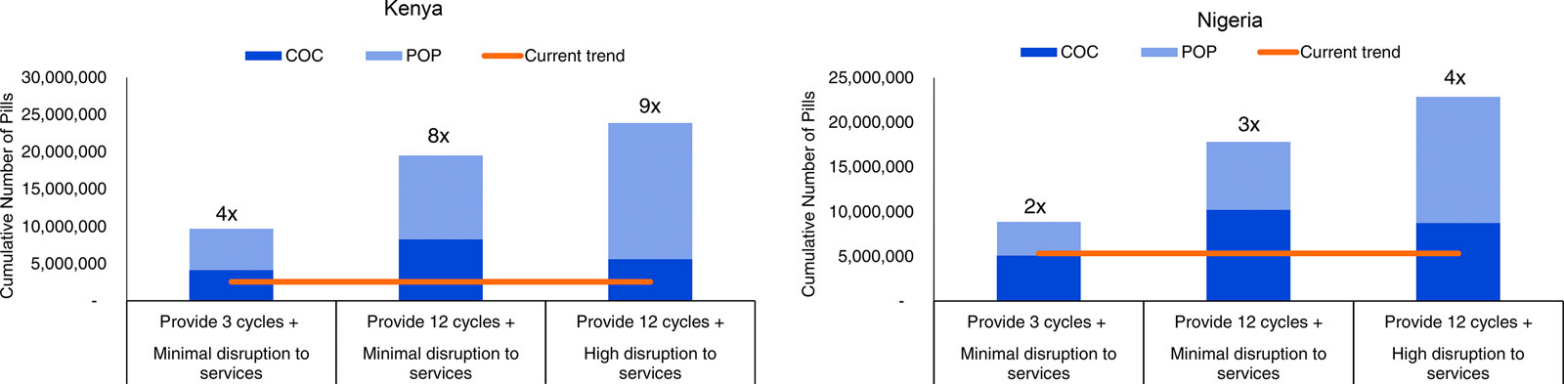
Cumulative Number of Pills Dispensed Over 6 months in Kenya and Nigeria Under Different COVID-19 Disruption Scenarios, April to September 2020 Abbreviations: COC, combined oral contraceptive pill; POP, progesterone-only pill.

The changes described would also have substantial implications for the supplies these countries will need in the short term. In both scenarios presented, we estimate a potential surge in demand for self-care contraceptive methods. Although the particular methods available and desirable will ultimately determine what is used, in our scenarios, much of the new demand for self-care methods comes from contraceptive pills. If in addition to the method switching described, advanced provision of methods is implemented, women would receive most of these commodities at once (i.e., front-loading distribution). With this combination in Kenya, pills dispensed could surge to between 4 times to 9 times the current levels over the coming 6 months, and in Nigeria the surge could be around half of these levels (Figure 2).

Another potential complication in satisfying an increased demand from women switching to pills would be the type of pill best suited to serve this “replacement” role. Certain institutions, such as the Royal College of Obstetricians and Gynecologists, have recommended the use of progesterone-only pills (POPs) or “mini-pills” as bridging methods because they have no contraindications for high blood pressure—in contrast with combined oral contraceptives—and thus do not require screening and are likely suitable for injectable users who are already receiving a progesterone-only product.^10^

As we look to a likely future where self-care methods fill gaps left by contractions in current service delivery options, we must also raise a critical concern around equity. Current data from the Commodity Gap Analysis reveal that self-care methods are already accessed disproportionately from the private sector—a disparity that may become even more stark if public sector providers find themselves overwhelmed in responding to COVID-19. This situation could mean that women who had previously accessed free or subsidized services through the public sector now find themselves struggling to pay out-of-pocket. The burden may be multiplied by advanced provision recommendations, or perhaps even more realistically, by the higher cost of revisits by women unable to purchase multiple months’ worth of supplies. These changes may coincide with broader economic disruptions in household income that limit women’s ability to pay. As we think about strategies to ensure continued access to contraception, we cannot forget this important factor. Potential mitigation options are discussed later, including financing approaches that can help ensure equitable access. These challenges are also explored further by Remme et al.^18^

As we look to a future where self-care methods may fill gaps left by contractions in current service delivery options, we must also raise a critical concern around equity.

## HOW DO WE ENSURE THAT COMMODITIES ARE AVAILABLE AND ACCESSIBLE TO WOMEN?

Although they are only illustrative, these 2 country scenarios in Kenya and Nigeria point to the potential impact of changes that may occur in the short term: decreased consumption of LARCs and provider-administered injectables offset by increased consumption of self-care methods as women seek alternatives that can be accessed with little or no face-to-face contact with service providers.

Each country’s response will differ as policy makers weigh the options before them, consider the realities of what supplies they already have on hand and what can be accessed in the near term, and observe the response from women as they find ways to continue to access contraception. We outline some key challenges and opportunities that should be considered regarding products and programs and clients.

### Challenges and Opportunities Ensuring Product Availability

Strategies to mitigate the impacts of COVID-19 can only be successful if there are supplies available to do so. Unpredictability and potentially rapid changes in demand will be challenging for centralized supply chains to respond to, especially as many of these changes run counter to recent trends. In the years leading up to the crisis, public sector procurement of pills declined to only 7% of couple-years of protection shipped in the public sector market in 2018.^14^ Public procurement of POP remains low,^19^ so stocks may be limited in countries. In addition, there are few World Health Organization prequalified pill manufacturers, especially for POPs.^20^ Continued sharing of accurate family planning supply data through existing channels with donors and manufacturers will be important, especially as we look to long-term supply needs.^21^

Near-term approaches that allow for flexibility and opportunism in responding to the ever-evolving landscape are needed. The need for increased procurement of self-care methods could prompt countries to engage with a wider set of generic manufacturers if quality can be assured. Countries could also look to leverage existing private, nongovernmental organizations and social marketing supply chains that work beyond the public sector, especially in contexts where these supply chains are already bringing large volumes of self-care methods into a country.

Near-term approaches that allow for flexibility and opportunism in responding to the ever-evolving supply chain landscape are needed.

Further, for countries and regions with domestic manufacturing, using these companies, if quality can be assured, may help reduce challenges with shipping and distribution of supplies. Member organizations of the Reproductive Health Supply Coalition’s ForoLAC (The Latin American and Caribbean Forum) are already working in Latin America to assemble a regional inventory of contraceptive manufacturers; similar initiatives in other regions could reduce the uncertainty of securing contraceptives manufactured “closer to home.”

In addition, donors and partners may need to explore opportunities for ensuring that actors across the supply chain have access to the working capital needed to keep these products moving. The likely temporary nature of these shift may bring special financing considerations.

### Challenges and Opportunities for Programs and Clients

Access to self-care methods are likely to come through private sector channels. These channels are well-placed to be nimble and often comprise large networks of pharmacies and shops that allow for easy access to self-care methods. However, this also presents challenges to ensure both equity and quality. Countries could explore strategies such as strategic purchasing, vouchers, and other financial support mechanisms to ensure that changes do not bring undue burdens, especially for the most vulnerable. Channeling subsidies directly to users could also support the development of more resilient and client centered contraceptive markets.^22^

We should also remember that women need more than just products. Telehealth and m-health services can play an important role in informing women’s choices over what contraceptives to use and answering any questions they may have. Where appropriate and feasible, countries may support use of the Standard Days Method, especially where the need for a physical product can be eliminated through phone apps. These changes will likely require effective communication efforts. Social and behavior change messages can reinforce help inform women about safe mitigation strategies, and address concerns over accessing services or switching methods.^23^

And of course, public sector service delivery will still play an important role, although that role may change. Using service delivery models that bring services closer to women and girls will be particularly important in light of mobility restrictions and concerns about interacting with health facilities. Community health workers, mobile outreach teams, and partnerships with small private health care providers could distribute free or subsidized products with more limited face-to-face contact.

Using service delivery models that bring services closer to women and girls will be important in light of mobility restrictions.

It is important to review procurement plans and programs developed before physical distancing to identify any mismatch with current reality and explore ways to meet current needs.

## THE WAY AHEAD

While focusing on short-term solutions to ensure accessibility during this global pandemic, we must also keep a focus on the long term. Many of the changes described will likely be *temporary*. So, as circumstances change and clinical services become more available, systems and supply chains must be in place to ensure that women can once again access the delivery and removal services not available during COVID-19.

We are entering a disruptive phase for essential health services—hopefully a comparatively short-lived phase—but one that could dramatically change both the content of national family planning programs and the ability of those programs to meet client needs. In the short term, it is important to review procurement plans and programs developed before physical distancing to identify any mismatch with current reality and explore ways to meet current needs. Now more than ever, we must bridge the often-siloed discussions about product and program to minimize the potentially devastating consequences of COVID-19 on women and girls around the world.

The analysis presented in this article provides a first look into quantifying potential shifts in contraceptive needs that could result from different service disruptions and mitigation strategies. An Excel-based tool, Modeling the Impact of COVID-19 on Reproductive Health Options (MICRO), has been developed that allows countries to replicate and expand the types of results shown here for Kenya and Nigeria. The tool integrates data from the Reproductive Health Supplies Visualizer^24^ on public sector shipments in recent years to contrast potential changes to recent trends. The model includes 2 scenarios aligned to results presented here; users can change the assumptions to explore their own custom mitigation scenarios. In addition, through the Global Family Planning Visibility and Analytics Network^25^ work is underway to assess risks from a supply chain perspective. We can only hope to mitigate the potentially devastating consequences of this global pandemic on women’s contraceptive choices if we focus on using data and collaborating to ensure that products and programs are moving together.

## Supplementary Material

20-00171-Supplemental_Table_clean.xlsx

## Notes

**Peer Reviewed**

**First published online:** June 19, 2020
